# Case series: cardiac sarcoma

**DOI:** 10.1093/ehjcr/ytad546

**Published:** 2023-11-07

**Authors:** Michael Killian, Timothy Barry, Carolyn Larsen, Said Alsidawi

**Affiliations:** Mayo Clinic Arizona, 5777 E. Mayo Blvd., Phoenix, AZ 85054, USA; Mayo Clinic Arizona, 5777 E. Mayo Blvd., Phoenix, AZ 85054, USA; Mayo Clinic Arizona, 5777 E. Mayo Blvd., Phoenix, AZ 85054, USA; Mayo Clinic Arizona, 5777 E. Mayo Blvd., Phoenix, AZ 85054, USA

**Keywords:** Sarcoma, Undifferentiated pleomorphic sarcoma, Round cell myxoid liposarcoma, Liposarcoma, Cardiac mass, Cardiac tumour, Cardiac malignancy, Case report, Case series

## Abstract

**Background:**

Cardiac masses encompass a wide differential including primary and secondary malignancies and can present with a variety of symptoms, many of which are non-specific. Early identification and classification are important, particularly for cardiac malignancies such as sarcomas as these are aggressive tumours with exceptionally poor prognoses when metastases are present at diagnosis.

**Case summary:**

We report two cases of patients who presented with dyspnoea and were diagnosed with cardiac sarcomas; the former a primary sarcoma (undifferentiated pleomorphic subtype) and the latter a secondary sarcoma (round cell myxoid liposarcoma) that serve as comparisons for presentation and management of different types of this disease. Computed Tomography (CT) and echocardiography imaging findings are demonstrated showing the typical location and morphology of each subtype.

**Discussion:**

Cardiac sarcomas are the most common primary cardiac malignancy, of which undifferentiated pleomorphic sarcoma is a common subtype. Undifferentiated pleomorphic sarcomas are aggressive, have a tendency to arise in the left atrium, and can appear similar to benign cardiac masses. Round cell myxoid liposarcomas by contrast are rare causes of secondary cardiac malignancies, metastasizing to the heart from soft tissues. Both diagnoses carry poor prognoses and although rare, are important to recognize as timely intervention with surgery, radiotherapy, and consideration of chemotherapy is key to maximizing survival.

**Learning points**

- Cardiac masses can present with a variety of symptoms, many of which are non-specific.
- Symptoms such as shortness of breath, syncope, and palpitations may predominate in the case of obstructive lesions whereas symptoms consistent with a transient ischaemic attack or stroke may be apparent in cases of lesion embolization. Constitutional symptoms such as fatigue, anorexia, and weight loss may also be present.
- Differential diagnoses for cardiac masses include primary and secondary malignancies, benign tumours, thrombi, vegetations, and other infective lesions such as tuberculoma/aspergilloma, calcific lesions, and miscellaneous cystic and embryonic remnants.
- Metastases to the heart greatly outnumber primary cardiac malignancies.
- Cardiac sarcomas are aggressive tumours and can arise both as primary malignancies or as metastases to the heart.
- Secondary cardiac malignancies, including sarcomas, typically arise in the right heart.
- Surgery is the mainstay of treatment for primary cardiac tumours with additional radiotherapy and chemotherapy for high-risk cases.
- Secondary cardiac sarcomas carry a poor prognosis for which palliative chemotherapy is often the only therapeutic option.

## Introduction

Cardiac masses encompass a wide range of differential diagnoses including primary and secondary malignancies and can present with a variety of symptoms, many of which are non-specific.^1^ Early identification and classification are important, particularly for cardiac malignancies such as sarcomas as these are aggressive tumours with exceptionally poor prognoses when metastases are present at diagnosis.^2^ We describe two cases of patients who presented with shortness of breath and were found to have cardiac masses. Their presentations, diagnostic work-up and management are outlined below along with imaging findings in each case that serve as comparisons between primary and secondary cardiac malignancies.

## Summary figure

### Summary table: features of primary vs. secondary cardiac sarcomas

**Table:** 

	Primary sarcoma	Secondary sarcoma
Most common subtype	Angiosarcoma	Leiomyosarcoma
Other frequent subtypes	Undifferentiated pleomorphic sarcoma (UPS) Leiomyosarcoma	Clear cell sarcoma Rhabdomyosarcoma
Location	Most frequently right atrium (angiosarcoma). Left atrium common site for other subtypes	Classically right atrium and right ventricle
Age at presentation	Typically 4th–6th decade of life	Variable
Presentation	Variable: non-specific symptoms, features of obstruction, embolism depending on size, location, and depth of invasion	
Metastases	Metastasize to lungs and mediastinal lymph nodes, kidney, skin, brain, and gastrointestinal tract	Can metastasize from a varied range of primary sarcoma sites
Management	Surgical resection for non-metastatic disease, ±neoadjuvant chemoradiotherapy	Palliative chemotherapy ± palliative surgical resection
Survival	Poor, ∼6–18 months	Exceptionally poor

## Patient 1

A 42-year-old Caucasian female presented to the emergency department with worsening shortness of breath over the past 3 weeks. This was associated with pleuritic chest tightness, a non-productive cough, light-headedness on stooping, and decreased oxygen saturations (SpO_2_ 70%) with exertion. She denied infective symptoms, such as fever and chills or history of tobacco use. COVID-19 PCR testing pre-admission was negative.

Her past medical history was significant for essential hypertension, obesity, cataracts, depression, and a hysterectomy. Her medications included albuterol prn, fluticasone prn, hydrochlorothiazide 25 mg o.d., loratadine 10 mg o.d., losartan 100 mg o.d., and meclizine 25 mg o.d. She was allergic to penicillin.

Her family history was non-contributory, she worked in healthcare, and alcohol consumption was 5 units per week. A review of systems was positive for mild headaches.

On examination, she required 1 L of oxygen to maintain oxygen saturations of 97%. Heart rate was 96 beats per minute, respiratory rate 24, and temperature 36.6°C. Blood pressure was 150/94 mmHg. Cardiovascular examination revealed regular rhythm and normal heart sounds with no appreciable murmur. Jugular venous distension and lower limb oedema were absent. She was not in respiratory distress. Coarse breath sounds were noted on the left side. Abdominal and neurological examinations were unremarkable.

Blood investigations are shown in *Table 1* below. The electrocardiogram (ECG) showed normal sinus rhythm at 95 b.p.m. and was otherwise unremarkable. A portable chest X-ray showed bilateral patchy infiltrates and computed tomography (CT) pulmonary angiography showed no evidence of pulmonary embolus but patchy ground-glass areas of opacification and septal thickening were present throughout the lungs bilaterally with small bilateral pleural effusions. A 5.5 × 3.7 cm mass was identified in the left atrium extending into the level of the mitral valve.

**Table 1:** Blood investigations

Component	Value	Ref range
White blood cell count	11.1	4.0–11.0 ×1000/UL
Haemoglobin	12.6	12.0–16.0 g/dL
Platelet count	197	130–450 K/UL
Absolute lymphocytes	1.5	1.0–3.2 K/μL
Absolute monocytes	0.7	0.3–1.1 K/μL
Absolute neutrophils	8.7	1.7–7.6 K/μL
Absolute eosinophils	0.1	0.0–0.5 K/μL
Absolute basophils	0.1	0.0–0.1 K/μL
Glucose	109	70–99 mg/dL
Urea nitrogen	14	6–20 mg/dL
Creatinine	0.9	0.5–0.9 mg/dL
Calcium	9.3	8.6–10.0 mg/dL
Sodium	136	136–145 mmol/L
Potassium	3.3	3.4–5.1 mmol/L
Chloride	99	98–107 mmol/L
Carbon dioxide	22	22–29 mmol/L
Estimated glomerular filtration rate (eGFR)	>59	>59 mL/min/1.73m^2^
Anion gap	15	3–11 mEq/L
Troponin T	<0.01	0.00–0.01 ng/mL
N-terminal pro-B-type natriuretic peptide	1026	0–167 pg/mL

Initially a broad differential was considered including congestive cardiac failure, respiratory tract infection, and reactive airway disease.

**Figure 1:**
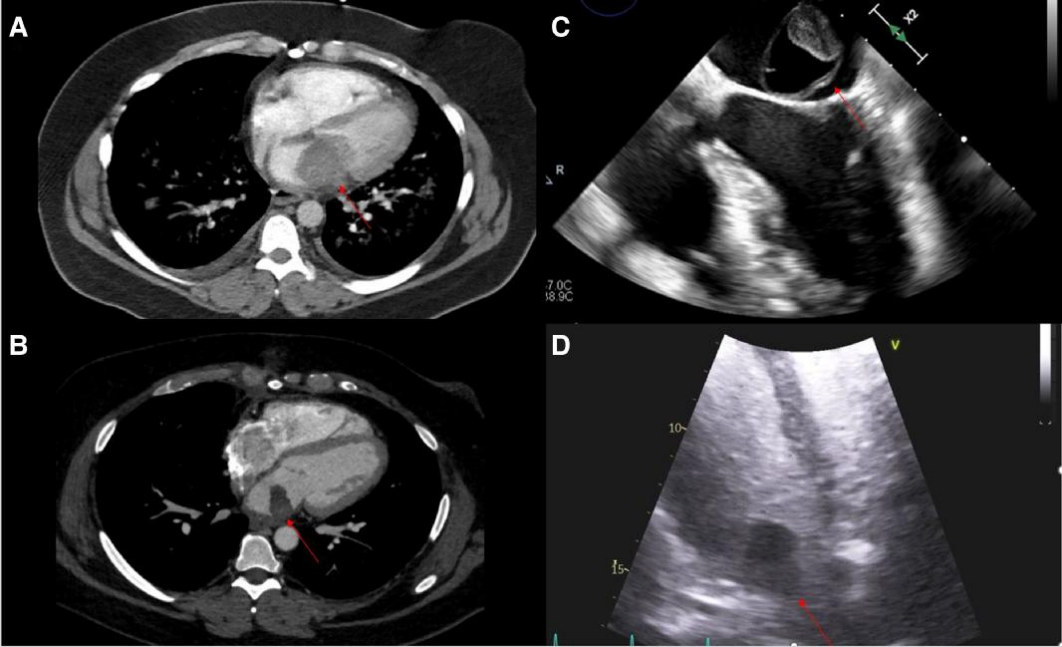
CT and echocardiographic images. (*A*) Initial CT pulmonary angiogram axial view demonstrating left atrial mass (arrow) and right pleural effusion. (*B*) Repeat CT pulmonary angiogram axial view 1 month later demonstrating recurrence of left atrial mass (arrow). (*C*) Intraoperative transoesophageal echocardiogram four-chamber view demonstrating left atrial mass. (*D*) Transthoracic echocardiogram apical four-chamber view demonstrating left atrial mass.

Her initial management included nebulizers, steroids, intravenous antibiotics, and diuretics, however her work of breathing increased and she developed type 2 respiratory failure requiring bilevel positive airway pressure ventilation. At this point, CT pulmonary angiography was obtained. Based on CT findings, she was commenced on a heparin infusion for possible left atrial thrombus and an urgent echocardiogram was ordered, revealing an enlarged left atrium and an echogenic structure attached to the mitral valve, impeding flow to the left ventricle. Ejection fraction was normal at 65–70% with mild concentric left ventricular hypertrophy. Right-sided function was normal. Imaging findings are shown in *Figure 1*.

Cardiothoracic opinion was sought, and she proceeded to emergent surgery. A left atrial mass, ∼5.5 cm in size, was resected, and a 600 mL serous right pleural effusion was drained. She had an uncomplicated post-operative recovery and was discharged on post-operative Day 4 with planned follow-up for pathology results. Additional medications on discharge included metoprolol, atorvastatin, and aspirin.

Histopathology of the mass revealed intimal cardiac sarcoma (undifferentiated pleomorphic cardiac sarcoma), high-grade [Fédération National de Lutte Contre le Cancer (FNCLCC) grade 3 of 3]. Oncology was involved, and a positron emission tomography (PET) scan was negative. She was referred for evaluation of possible positive surgical margins to another centre. One month later as part of this evaluation, she underwent a repeat CT chest and echocardiogram. At this time, she was progressing well with cardiac rehabilitation without recurrence of symptoms. The echo again revealed a mobile left atrial mass attached to the lateral wall of the left atrium, extending along the posterior mitral valve leaflet. CT confirmed these findings, demonstrating a 27 × 24 × 18 mm mass with heterogenous internal enhancement.

A heparin infusion was commenced due to high suspicion of atrial thrombus, and cardiothoracic opinion was sought once again. She proceeded to a redo sternotomy where a 3 cm diameter left atrial mass was excised. She had an uncomplicated post-operative course and was discharged on warfarin. Histopathology confirmed recurrent sarcoma, and she underwent four cycles of chemotherapy (doxorubicin, ifosfamide). Her post-treatment PET scan revealed a right ovarian mass that was resected. Histopathology confirmed metastatic disease. Over the following weeks, she presented with a small bowel obstruction secondary to metastases requiring further surgery and also suffered a left hemisphere stroke secondary to embolic left internal carotid artery occlusion. She was found to have a third recurrence of a 2 × 3 cm left atrial mass and succumbed to her disease weeks later.

## Patient 2

A 67-year-old Caucasian male was transferred from an outside centre with acute hypoxic respiratory failure for further work-up. He had presented to an outside centre with a 4-week history of increasing shortness of breath. A chest X-ray obtained at the onset of symptoms in the community was non-diagnostic. He denied any orthopnoea or paroxysmal nocturnal dyspnoea and had no lower limb oedema. He had no chest pain and denied cough, sputum, wheeze, or infective symptoms.

His past medical history was significant for round cell myxoid liposarcoma diagnosed 17 years prior localized to his right foot for which he had undergone a right below knee amputation. His course had been complicated by recurrence 4 years later in the left flank that required resection and a further re-excision for positive margins. He received radiotherapy that was complicated by peripheral vascular disease to his right thigh requiring angioplasty and stenting. He had been in remission since with negative imaging 4 months prior to his current presentation. He had a deep venous thrombosis to his right stump for which he was anticoagulated, hypertension, hyperlipidaemia, and an indeterminate right renal mass for which he underwent ablation 5 years prior to this admission. He was under surveillance for multiple bilateral pulmonary nodules that had been stable in size on CT imaging 4 months prior.

His medications included apixaban 5 mg b.i.d., aspirin 81 mg o.d., ezetimibe 10 mg o.d., and lisinopril 20 mg o.d. He was allergic to moxifloxacin.

His family history was non-contributory. He lived with his wife and had given up tobacco in the 1980s. He consumed on average 20 units of alcohol per week.

On examination, he was in respiratory distress, hypoxic, and tachypnoeic. Oxygen saturations were in the 80s despite maximal bilevel positive airway pressure settings. His respiratory rate was 25, heart rate 98 beats per minute, blood pressure 145/105 mmHg, and temperature 36.4°C. On cardiovascular examination, his pulse was regular, with normal heart sounds and no murmurs. Jugular venous distension and peripheral oedema were absent. His respiratory examination revealed normal breath sounds without crackles or wheezes. His abdominal, musculoskeletal, and neurological examinations were unremarkable apart from expected findings given his prior surgical interventions.

**Figure 2:**
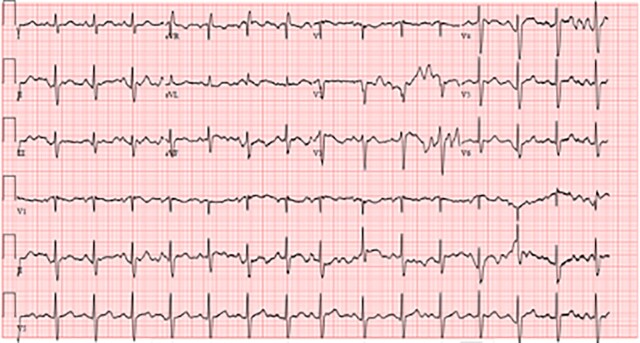
Admission ECG.

His admission ECG (*Figure 2*) showed sinus tachycardia with a positive R wave in aVR. His blood investigations and arterial blood gas results are shown in *Tables 2* and *3*. A respiratory viral panel was negative for COVID-19, influenza, or respiratory syncytial viral infections. His chest X-ray was non-diagnostic.

**Table 2:** Blood investigations

Component	Value	Ref range
White blood cell count	8.3	3.4–9.6 × 10(9)/L
Haemoglobin	18.8	13.2–16.6 g/dL
Erythrocytes	5.72	4.35–5.65 × 10(12)/L
Haematocrit	55.3	38.3–48.6%
Mean corpuscular volume	96.7	78.2–97.9 fL
Red blood cell distribution width	14.9	11.8–14.5%
Platelet count	151	135–317 × 10(9)/L
Neutrophils	6.29	1.56–6.45 × 10(9)/L
Lymphocytes	1.31	0.95–3.07 × 10(9)/L
Monocytes	0.60	0.26–0.81 × 10(9)/L
Eosinophils	0.01	0.03–0.48 × 10(9)/L
Basophils	0.04	0.01–0.08 × 10(9)/L
Glucose	95	70–140 mg/dL
Urea nitrogen	12.6	8–24 mg/dL
Creatinine	1.15	0.74–1.35 mg/dL
Calcium	10.0	8.8–10.2 mg/dL
Sodium	141	135–145 mmol/L
Potassium	4.4	3.6–5.2 mmol/L
Chloride	108	98–107 mmol/L
Estimated glomerular filtration rate (eGFR)	70	>60 mL/min/BSA
Troponin T	17	≤15 ng/L
N-terminal pro-B-type natriuretic peptide	205	0–167 pg/mL
Bilirubin (total)	1.6	≤1.2 mg/dL
Bilirubin (direct)	0.3	0.0–0.3 mg/dL
Aspartate aminotransferase (AST)	29	8–48 U/L
Alanine transaminase (ALT)	26	7–55 U/L
Alkaline phosphatase	110	40–129 U/L
Albumin	4.6	3.5–5.0 g/dL
Procalcitonin	0.06	≤0.08 ng/mL
Thyroid stimulating hormone	3.9	0.3–4.2 mL U/L

**Table 3:** Arterial blood gas on 100% oxygen

Component	Value	Ref range
pH	7.443	7.35–7.45
pCO_2_	18.7	35–45 mmHg
pO_2_	43	75–100 mmHg
Bicarbonate	12.8	22–26 mmol/L
Oxygen saturation	83	95–100%

CT angiography of the chest was negative for pulmonary embolus but showed a large ∼5.7 cm diameter intraventricular mass in the right ventricle displacing the intraventricular septum. Differentials included malignancy or thrombus. Multiple right-sided pulmonary nodules were also noted, suspicious for pulmonary metastases.

**Figure 3:**
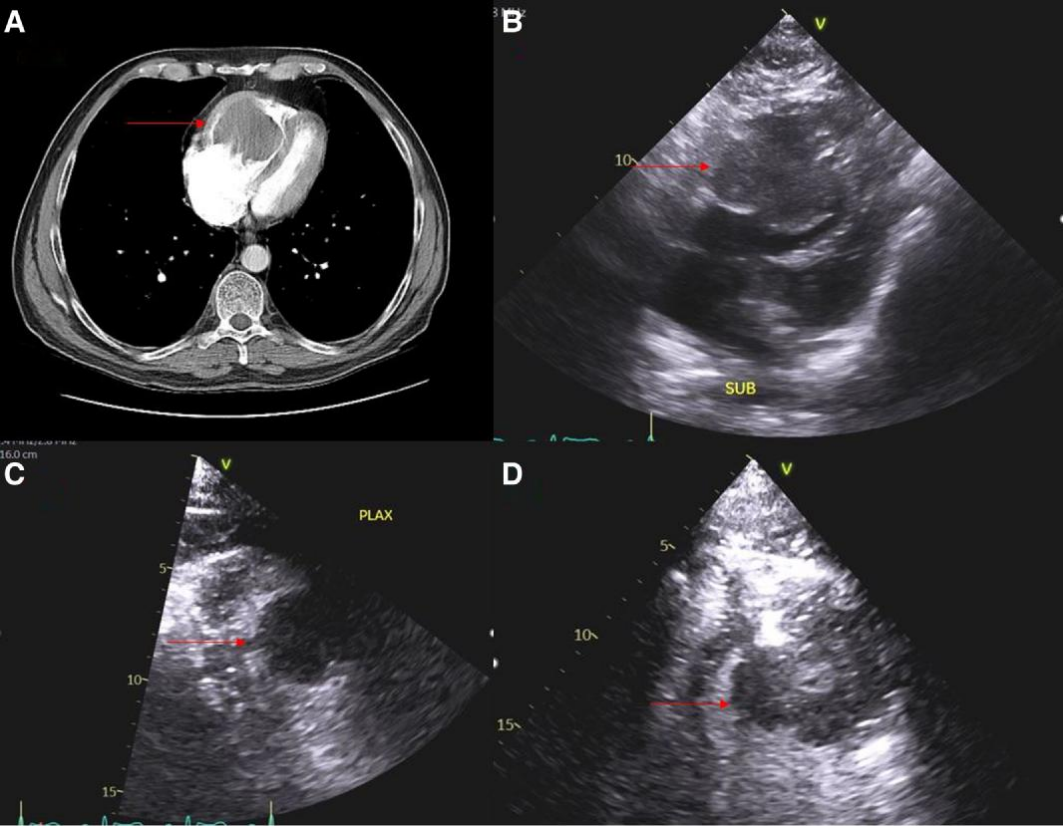
CT and echocardiographic images. (*A*) CT pulmonary angiogram axial view demonstrating large right ventricular mass (arrow) with septal displacement. (*B*) Transthoracic echocardiogram subcostal view corresponding to CT image demonstrating right ventricular mass (arrow) compressing left ventricle. (*C*) Transthoracic echocardiogram parasternal long axis view with imaging enhancer showing right ventricular mass. (*D*) Transthoracic echocardiogram apical four-chamber view with imaging enhancer demonstrating right ventricular mass (arrow).

His echocardiogram revealed a 7.9 × 5.3 cm well-defined mass occupying the right ventricle from the base to the apex. His left ventricular function was normal with a normal ejection fraction. A large non-restrictive atrial septal defect (2.6 cm) was also noted. There was no pericardial effusion. Imaging findings are shown in *Figure 3*.

He remained hypoxic despite maximal non-invasive ventilatory support with oxygen saturations persisting in the 80s. A high anion gap metabolic acidosis driven by a high lactate (3.4) and starvation ketosis was noted. Given his atrial septal defect, positive pressure ventilation was avoided and he was transitioned to high-flow nasal cannula. Oxygen saturations were maintained at 80 with 100% FiO_2_ at 60 L per minute. A multidisciplinary team involving critical care, cardiology, cardiothoracic surgery, oncology, and palliative medicine was involved in the patient’s care. On review of his echocardiogram and CT imaging, a presumptive diagnosis of recurrent round cell myxoid liposarcoma with cardiac and pulmonary metastases was made. The definitive diagnosis requiring cardiac or lung biopsy and further work-up for which the patient was too unwell to undergo, a shared decision was reached between the multidisciplinary team and the patient to pursue hospice care and he was transferred home with palliative measures.

## Discussion

Of all primary cardiac tumours, typically over 90% are benign, the most common of which in adults is papillary fibroelastoma, followed by atrial myxoma, the most common left atrial tumour.^3,4^ The remaining 10% are malignant tumours, either falling into the sarcoma or lymphoma categories, with sarcomas predominant.^5^

Differential diagnoses include primary and secondary malignancies, benign tumours, thrombi, vegetations, and other infective lesions such as tuberculoma/aspergilloma, calcific lesions, and miscellaneous cystic and embryonic remnants to name but a few.^6^ Where primary cardiac tumours are suspected, T1 and T2 weighted cardiac MRI with late gadolinium enhancement can be diagnostic.^7^ Notably in the above cases, cardiac MRI was not performed. Both patients underwent CT imaging in the first instance due to acute hypoxic presentations. The need for surgery with an expectant histopathological diagnosis in the case of Patient 1, and the decision to proceed with palliative management on diagnosis in Patient 2, precluded the additional utility of cardiac MRI in these cases.

Primary cardiac sarcomas include cardiac angiosarcoma, UPS, and cardiac leiomyosarcoma. Undifferentiated pleomorphic sarcoma makes up ∼30% of cases and is the second most common subtype after cardiac angiosarcoma.^5,8^

Undifferentiated pleomorphic sarcomas are aggressive tumours, rapidly infiltrating all layers of the heart before invading adjacent mediastinal structures and metastasizing. Metastases are typically present in 80% of cases at diagnosis, precluding resection and associated with poor prognoses. Survival is typically weeks to months.^8^

Classically they arise in the left atrium, without sex predilection, between the 4th and 6th decades.^5^ Dyspnoea, chest pain, weight loss, and malaise are common presenting symptoms. Patients may also present with metastatic disease, common sites include lung, kidney, and skin.

Undifferentiated pleomorphic sarcoma can simulate myxoma both in clinical presentation and macroscopically on imaging but unlike myxoma, it may form multiple masses. It can descend from the atrium, cause impingement on the mitral valve, and extend into the pulmonary veins.^8^ It is typically a diagnosis of exclusion because as its name implies, UPS has no areas of specific tissue differentiation by light microscopic appearance or immunohistochemistry.^5^

Grading is according to the FNCLCC system, a cumulative score involving tumour differentiation, mitotic count, and tumour necrosis.

The mainstay of treatment is surgical resection if possible. Location and degree of infiltration can make complete excision with achievement of adequate margins particularly challenging. Implantation of an artificial heart, total heart removal with *ex vivo* repair on the bench, followed by autotransplantation, and orthotopic transplantation can be considered on a case by case basis in the absence of metastases.^9^ Neoadjuvant radiotherapy may be considered pre-operatively. Adjuvant chemotherapy including anthracyclines such as epirubicin and doxorubicin in addition to ifosfamide has been recommended in high-risk cases to improve survival.^8^ Where patients present advanced or metastatic disease, treatment consists of chemoradiotherapy and palliative care.

Metastases to the heart, or secondary cardiac tumours, typically outnumber primary cardiac tumours by a factor of 20 to 1.^10^ Common primaries include lung, breast, kidney, liver, and lymphomas. They exhibit predilection for the right atrium and ventricle. Clinical presentation follows that of the majority of cardiac masses, with features of obstruction, embolism, and systemic symptoms depending on size, location, and degree of invasion.

Soft tissue sarcomas have an overall incidence of 4–5 per 100 000/year of which liposarcomas are the most common subtype.^11^ Liposarcomas are further subdivided into multiple groups, of which round cell myxoid is responsible for 30% of cases, after the differentiated and dedifferentiated liposarcoma subtypes.^12^

The proportion of the round cell component in addition to tumour size determines prognosis, with more than 5% round cells indicative of a worse prognosis.^13,14^ They typically arise in the deep tissues of the extremities and metastasize to other soft tissues, bone, the chest wall, and the abdominal cavity, particularly the retroperitoneum.^15^ Cardiac metastases are rare. Surgery and radiation are the mainstays of treatment. Liposarcomas are generally poorly responsive to chemotherapy however better responses are seen in round cell myxoid.^16^ Doxorubicin, ifosfamide, and newer apoptotic agents such as trabectedin may be used. Approximately 20% of patients will experience metastases within 5 years.^14^

In terms of the cases illustrated, Patient 1 proceeded to emergent surgery for resection of the initial obstructive mass. In the intervening 4-week period from histological confirmation of sarcoma (with a negative PET scan) and tertiary centre evaluation, she was found to have a recurrence, which was again resected followed by adjuvant chemotherapy. The short time period of this recurrence followed by the further recurrences and embolic event demonstrates the aggression and morbidity associated with UPS.

By contrast, the time elapsed between initial round cell myxoid liposarcoma diagnosis in Patient 2 and cardiac metastasis spanned years however he also presented in extremis with a high burden of disease in which case disease modifying treatment was inappropriate.

These cases serve as illustrations for the presentation and management of primary and secondary sarcomas and the associated morbidity and mortality that exist with sarcomas of these particular subtypes.

## Data Availability

The data pertaining to this case series can be made available on request.

## Lead author biography

Dr Michael Killian is a graduate of UCC, Cork, Ireland who is pursuing a career in Cardiology.

**Consent:** Patient consent for publication of both cases was obtained as per COPE guidelines.

**Funding:** No funding was received in relation to this manuscript.
